# Trends and Hotspots in Nanoparticles for the Targeted Delivery of Nucleic Acids: A Ten-Year Bibliometric Study

**DOI:** 10.3389/fphar.2022.868398

**Published:** 2022-05-04

**Authors:** Yingzhao Huang, Qi Zhan, Chenzhou Wu, Nailin Liao, Zhou Jiang, Haoran Ding, Kunyu Wang, Yi Li

**Affiliations:** State Key Laboratory of Oral Diseases, National Clinical Research Center for Oral Diseases, West China Hospital of Stomatology, Sichuan University, Chengdu, China

**Keywords:** nanoparticles, targeted delivery, exosomes, genome editing, bibliometrics, CiteSpace

## Abstract

Nanoparticles for the gene therapy field have seen remarkable progress over the last 10 years; however, low delivery efficiency and other reasons impede the clinical translation of nanocarriers. Therefore, a summary of hotspots and trends in this field is needed to promote further research development. In this research, from 2011 to 2021, 1,221 full records and cited references of Web of Science–indexed manuscripts regarding nanoparticle-targeted delivery systems have been analyzed by CiteSpace, VOSviewer, and MapEquation. In these software, keywords co-occurrence networks, alluvial diagram, co-citation networks, and structural variation analysis were carried out to emphasize the scientific community’s focus on nanomedicine of targeted delivering of nucleic acids. Keywords such as transfection efficiency, tumor cell, membrane antigen, and siRNA delivery were highlighted in the density map from VOSviewer. In addition, an alluvial flow diagram was constructed to detect changes in concepts. In the co-citation network, cluster 1 (exosomes) and cluster 17 (genome editing) were new research fields, and the efforts in modifying nanoparticles were revealed in the structural variation analysis. Aptamer and SELEX (systematic evolution of ligands by exponential enrichment) represented a helpful system in targeted delivery. These results indicated that the transfection efficiency of nanocarriers required continuous improvements. With the approval of several nucleic acid drugs, a new content of nanoparticle carriers is to introduce gene-editing technology, especially CRISPR/Cas9 (clustered regularly interspaced short palindromic repeats/CRISPR–associated protein 9). In addition, exosomes have great potential as targeted nanoparticles. By mapping the knowledge domains of nanomedicine in targeted delivering of nucleic acids, this study analyzed the intellectual structure of this domain in the recent 10 years, highlighting classical modifications on nanoparticles and estimating future trends for researchers and decision-makers interested in this field.

## Introduction

Despite significant advances in gene medicine, translating the results from research to the clinic to treat both genetic disorders and acquired diseases by introducing nucleic acids is still a challenging task (Wang et al., 2019). To date, more than 900,000 articles on the topic of gene therapy can be retrieved from the Web of Science; meanwhile, only around 3,180 gene therapy clinical trials have been approved, ongoing or completed worldwide, and 56.4% of these are in phase I^1^. Suitable vectors prevent naked nucleic acids introduced into the body from ubiquitous nucleases and renal elimination, and facilitate the process of targeted delivery and efficient internalization (Ren et al., 2021). Nonviral carriers, especially nanoparticles, have demonstrated tremendous potential in the targeted delivery of genetic material in treating hereditary transthyretin amyloidosis, pancreatic cancer, and other diseases with lower immunogenicity, with larger loading capacity, and being free from unexpected gene integration when compared to viral vectors (Ohno et al., 2013; Kamerkar et al., 2017; Adams et al., 2018; Yan et al., 2019; Cheng et al., 2020; Li et al., 2021). Nanotechnology is widely used in the medical industry and forms an important class of drug and nucleic acid delivery systems to accelerate the effect of drugs in the human body (Ravindran et al., 2018). Although various nanoparticles, such as lipid complexes, gold nanoparticles, and extracellular vesicles, have been extensively developed in recent years, low delivery efficiency, potential cytotoxicity, and immaturity for their application limit the steps to translation (Liu et al., 2017; Amreddy et al., 2018). Therefore, nano-delivery systems for nucleic acid are worthy of further research to meet the growing demands in nano-medicine and gene therapy translations.

Bibliometrics is a branch of informatics that evaluates the current situation and future research development trends, and professional software mapping knowledge domains may form an intuitive result. Before visualizing the map, data must be retrieved from the database, processed to transform, clustered to reduce dimensions, and organized as a network that contains nodes and links. Depending on the different items of interest and their relationships, networks are divided into co-occurrence networks, co-citation networks, and collaboration networks, which allow keyword–keyword, reference–reference, or author–author connections to uncover clues in the research field. Keywords in the same article tend to have a relationship in certain discipline and thus can be connected with links to form a co-occurrence network; analogously, references cited by the same article are connected for a co-citation network. CiteSpace is a software famous for excellently performing co-citation analysis and displaying the trends and developments in scientific domains (Chen, 2006; Chen, 2013). In addition, the density view provided by VOSviewer has a fantastic visualization in the co-occurrence network (Moral-Munoz et al., 2020). The alluvial flow diagram, presented by the MapEquation—a web-based application, shows the change of the network over time, which intuitively indicates the tendency of fields. By combining bibliometrics with data visualization, the intellectual structure of a specific domain can be established quantitatively and graphically for evaluating literature performance, detecting central issues, and breaking discipline dilemmas.

We researched and analyzed the intellectual structure of the nanoparticles targeted to deliver nucleic acids, summarized the hotspots of nanoparticles for gene therapy, and identified research trends to provide clues, ideas, and information for researchers and decision-makers interested in this field.

## Materials and Methods

The data were retrieved from the Web of Science Core Collection (SCI-EXPANDED, SSCI, A&HCI, CPCI-S, CPCI-SSH, ESCI, CCR-EXPANDED, and IC) with Retrieval Strategy 1 (see the Retrieval Strategy 1 section at the end of article) and dated from 1 January 2011 to 29 August 2021. A total of 1,232 full records and cited references were exported to CiteSpace 5.8.R2 (64-bit) to remove the duplicates, editorials, and proceedings papers; only 1,221 articles and reviews remained for further analysis according to the CiteSpace manual (Chen, 2014). Time slicing was performed from January 2011 to December 2021 for 1 year per slice, which meant that the data were sliced by year from 2011 to 2021 to generate 11 networks. The top 50 most frequently cited or occurring items were selected each year, the Pathfinder Algorithm was used to prune unnecessary links, then the 11 pruned networks were merged into a final network (Chen and Morris, 2003). The networks were viewed in CiteSpace, and the network information was exported to Microsoft Excel 2019 or other software when necessary. Modularity was used to evaluate the quality of the networks. Considering that a network contains several communities (clusters), modularity investigates the connection between them. High modularity means that the communities are well distinguished from one another (Chen, 2012). The modularity (Q) of an exemplary network varies from 0.3 to 0.7 or higher (Newman and Girvan, 2004).

### Keywords Co-Occurrence Networks

In CiteSpace, the top 50 most frequently used keywords were extracted as nodes annually, while links connected keywords when they co-occurred in one or more articles. The merged network was exported in Pajek (.net) format and displayed in VOSviewer 1.6.17 following the manufacturer’s instructions (van Eck, 2021). To generate an alluvial diagram, the networks were exported separately by year from CiteSpace in .net format, loaded and simplified successively in the alluvial generator^2^ (Bohlin et al., 2014).

### Co-Citation Networks

Before proceeding to the co-citation networks, two definitions had to be clarified. First, unless otherwise specified, the citing articles refer to the 1,221 articles and reviews that remain after removing duplicates, editorials, and proceedings papers, while the cited references are literature cited by these 1,221 records. The publication year of the citing articles ranges from 2011 to 2021, while the publication date of the cited references naturally can be traced back to an earlier period.

The co-citation network consisted of the top 50 most frequently cited references every year, and the references cited by the same article were linked. After measuring the co-citation similarities by cosine coefficients, a hard clustering approach was adopted to generate non-overlapping clusters, and further information can be found in the work by Chen et al. (2010). Specially, the value of silhouette shows the homogeneity of a particular cluster; a credible silhouette score tends to be close to 1 in general (Rousseeuw, 1987; Chen, 2013). After clustering the cited references by similarity, the structure of a co-citation network can be interpreted as the cognitive organization of science, and the citing articles are research front based on this intellectual foundation (Persson, 1994; Chen, 2006). Different clusters in CiteSpace were named with keywords extracted from the citing reference by the log-likelihood ratio algorithm.

A structural variation analysis (SVA) of the co-citation network was also conducted to discover the citing articles those profoundly influenced the modularity of the clusters. As scientific knowledge develops, the co-citation network structure changes. When a given citing article is introduced, it adds links to the co-citation network and changes the intellectual structure. By evaluating the change degree of the structure of a baseline network, the potential value of a certain citing article can be predicted. The change degree is appraised by the modularity change rate (MCR) as shown in the previous description from Chen (2012):

$$\text{MCR}\left(\text{a}\right)=\frac{\text{Q}\left(G_{\text{baseline}},\text{C}\right)-\text{Q}\left(G_{\text{baseline}⊕\text{Ga}},\text{C}\right)}{\text{Q}\left(G_{\text{baseline}},\text{C}\right)}\times 100$$

In this formula, Q(G_baseline_, C) represents the modularity of the baseline network *G* partitioned by a partition *C*, and after the citing article *a* is added to the network, the modularity is changed to Q(G_baseline⊕Ga_, C). The MCR(a) evaluates how deep the baseline network *G* is changed by introducing citing article *a*. Generally, a review article changes the structure more than a research article and holds a higher MCR owing to comprehensive conception and knowledge (Chen, 2012).

**FIGURE 1:**
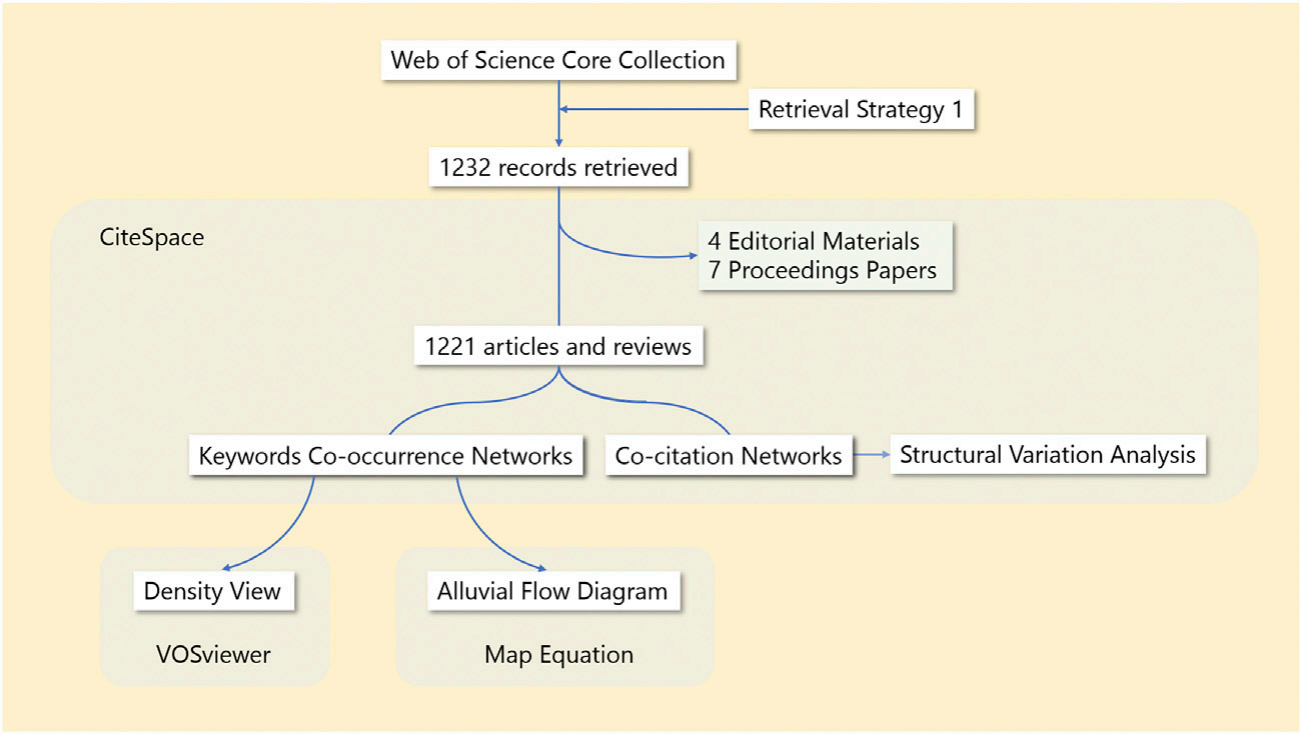
The workflow of our study.

The workflow of this study is presented in Figure 1.

## Results

### A Glimpse on Keywords Co-Occurrence Network

**FIGURE 2:**
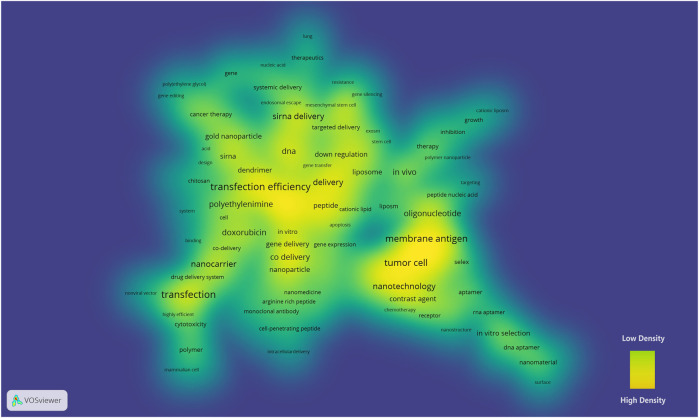
Density map of the keyword co-occurrence network of nanoparticles in nucleic acid–targeted delivery. A higher density represents a closer distance between the nodes and a larger number of neighboring nodes. The color of the map varies from yellow (high density) to green (low density) (van Eck, 2021).

A merged network that consisted of 172 nodes and 213 links (modularity Q = 0.7922) was exported to VOSviewer and shown as a density view (Figure 2). Keywords such as transfection efficiency (frequency = 35), tumor cell (frequency = 2), membrane antigen (frequency = 2), siRNA delivery (frequency = 154), and nanotechnology (frequency = 51) had a high density in the density map.

### Detect Trends and Hotspots in the Co-Citation Network

**FIGURE 3:**
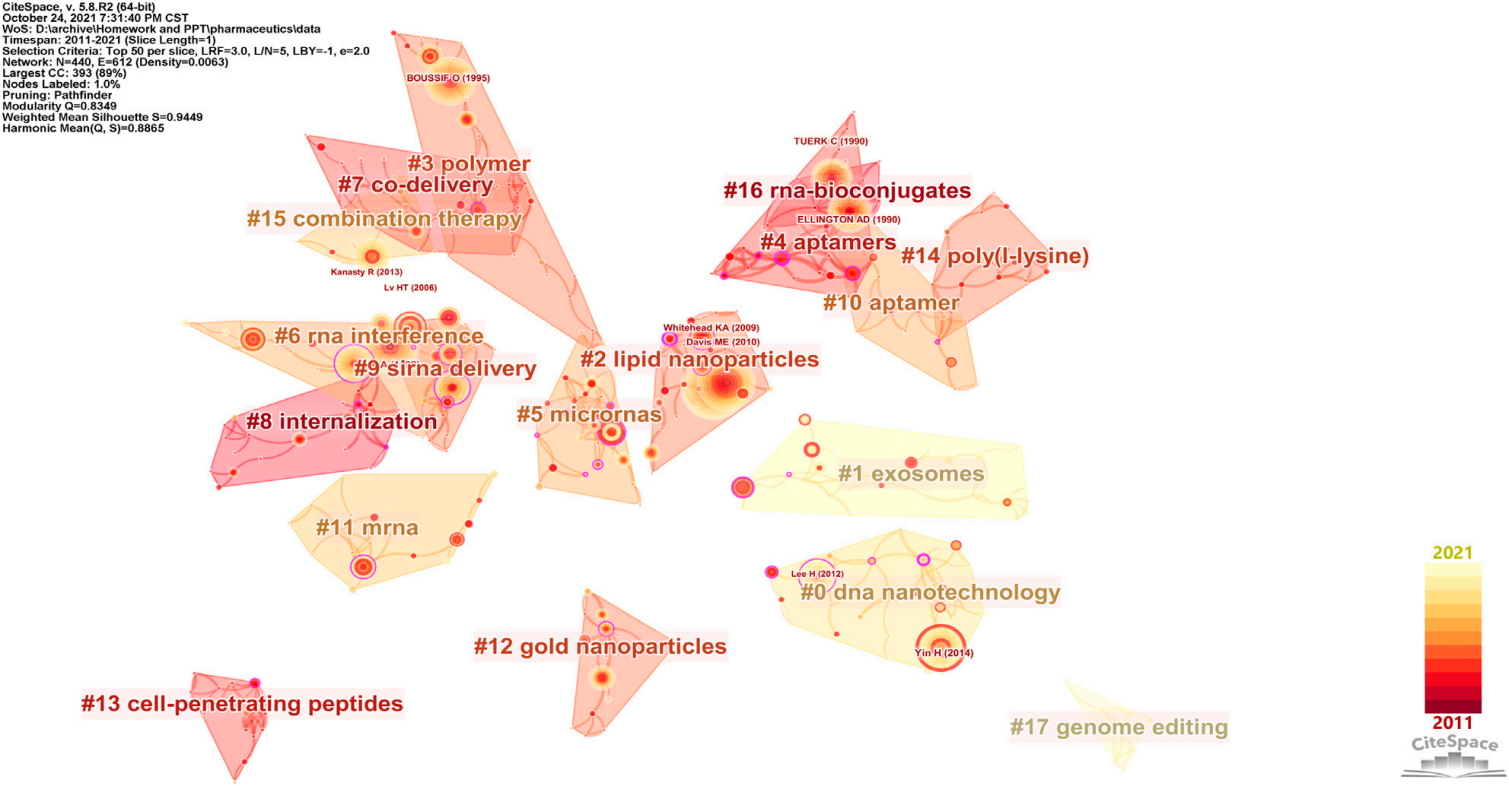
Co-citation network of nanoparticles in nucleic acid–targeted delivery. In this map, the color varied from dark red to light yellow and represented the time slice from 2011 to 2021. References were drawn as nodes with the citation tree rings, and the thickness of the tree rings was directly proportional to the frequency of the articles. Clusters are shown as blocks colored by the average number of citing years, numbered by size (#0 = largest, #17 = smallest), and labeled by keywords of the citing articles (log-likelihood ratio).

The co-citation network contained 440 cited references and 612 links (Q = 0.8349), with 18 clusters numbered from #0 to #17 that correspond to the cluster sizes from the largest to the smallest (Figure 3). Cluster #1 exosomes are considered a research hotspot regarding both the mean publication year and the mean citing year (Table 1). Although the average publication year of cluster #17 genome editing was 2013, the citation events occurred mainly in 2021 (Table 1, Supplementary Table S1), indicating that genome editing has recently been an active field. The details of cluster #17 are shown in Supplementary Table S1. In the theory of the structural variation model provided by Chen (2012), creative work usually accepts intellectual diversity while deeply changing the citation network. Generally, the review articles are prone to present a higher structural variation rate due to the considerable quantity of references and condensation of concepts. Thus, to emphasize the advanced original studies in their respective domains, review articles were eliminated and research articles with a high modularity change rate (MCR) were selected as shown in Table 2, which were all related to the further development of new nanomaterials.

**TABLE 1:** Cluster information of the co-citation network of nanoparticles in nucleic acid–targeted delivery.

Cluster ID	Size	Silhouette^a^	Mean publishing year	Mean citing year	Label (log-likelihood ratio, p-level)
0	37	0.94	2012	2018	DNA nanotechnology (17.65, 1.0E-4)
1	31	0.98	2015	2020	Exosomes (13.27, 0.001)
2	30	0.96	2008	2015	Lipid nanoparticles (5.51, 0.05)
3	28	1	2005	2015	Polymer (7.63, 0.01)
4	28	1	2005	2014	Aptamers (27.01, 1.0E-4)
5	26	0.83	2009	2017	Micro RNAs (6, 0.05)
6	26	0.94	2009	2016	RNA interference (8.7, 0.005)
7	23	0.94	2006	2014	Co-delivery (13.89, 0.001)
8	23	0.98	2007	2013	Internalization (8.91, 0.005)
9	20	0.93	2007	2015	siRNA delivery (5.63, 0.05)
10	19	0.88	2010	2016	Aptamer (12.39, 0.001)
11	18	0.84	2008	2017	mRNA (6.45, 0.05)
12	17	0.98	2009	2015	Gold nanoparticles (7.62, 0.01)
13	16	1	2008	2014	Cell-penetrating peptides (15.26, 1.0E-4)
14	16	0.92	2010	2015	Aptamer (11.61, 0.001)^b^
15	13	0.96	2010	2017	Combination therapy (8.55, 0.005)
16	13	0.95	2009	2013	RNA- bioconjugates (6.63, 0.05)
17	9	1	2013	2021	Genome editing (23.91, 1.0E-4)

a The value of silhouette shows the homogeneity of a particular cluster; a credible silhouette score tends to be close to 1 in general.

b In Figure 3, cluster #14 was labeled poly(l-lysine) by using a mutual information algorithm, which is speculated to be a bug or special setting of CiteSpace.

**TABLE 2:** Top 10 original studies with the highest modularity change rate (MCR) from 2011 to 2021.

Cited frequency	MCR	Year	Title
71	98.81	2013	Site-Specific Antibody–Polymer Conjugates for siRNA Delivery
21	97.33	2013	Highlighting the Role of Polymer Length, Carbohydrate Size, and Nucleic Acid Type in Potency of Glycopolycation Agents for pDNA and siRNA Delivery
62	96.94	2014	Exosome-Encased Spherical Nucleic Acid Gold Nanoparticle Conjugates As Potent MicroRNA Regulation Agents
0	96.65	2020	Peptide Spiders: Peptide–Polymer Conjugates to Traffic Nucleic Acids
24	94.44	2013	Hydrophobic and Membrane-Permeable Polyethylenimine Nanoparticles Efficiently Deliver Nucleic Acids *In Vitro* and *In Vivo*
21	92.71	2015	Targeted Decationized Polyplexes for siRNA Delivery
46	89.81	2014	Probing the Inherent Stability of siRNA Immobilized on Nanoparticle Constructs
1	86.84	2021	Evaluation of Dendronized Gold Nanoparticles as siRNAs Carriers into Cancer Cells
20	85.18	2013	Targeted Gene Delivery With Noncovalent Electrostatic Conjugates of sgc-8c Aptamer and Polyethylenimine
6	84.56	2017	Efficient *In Vivo* siRNA Delivery by Stabilized D-Peptide–Based Lipid Nanoparticles

### Alluvial Flow Diagram: Change of Concepts

**FIGURE 4:**
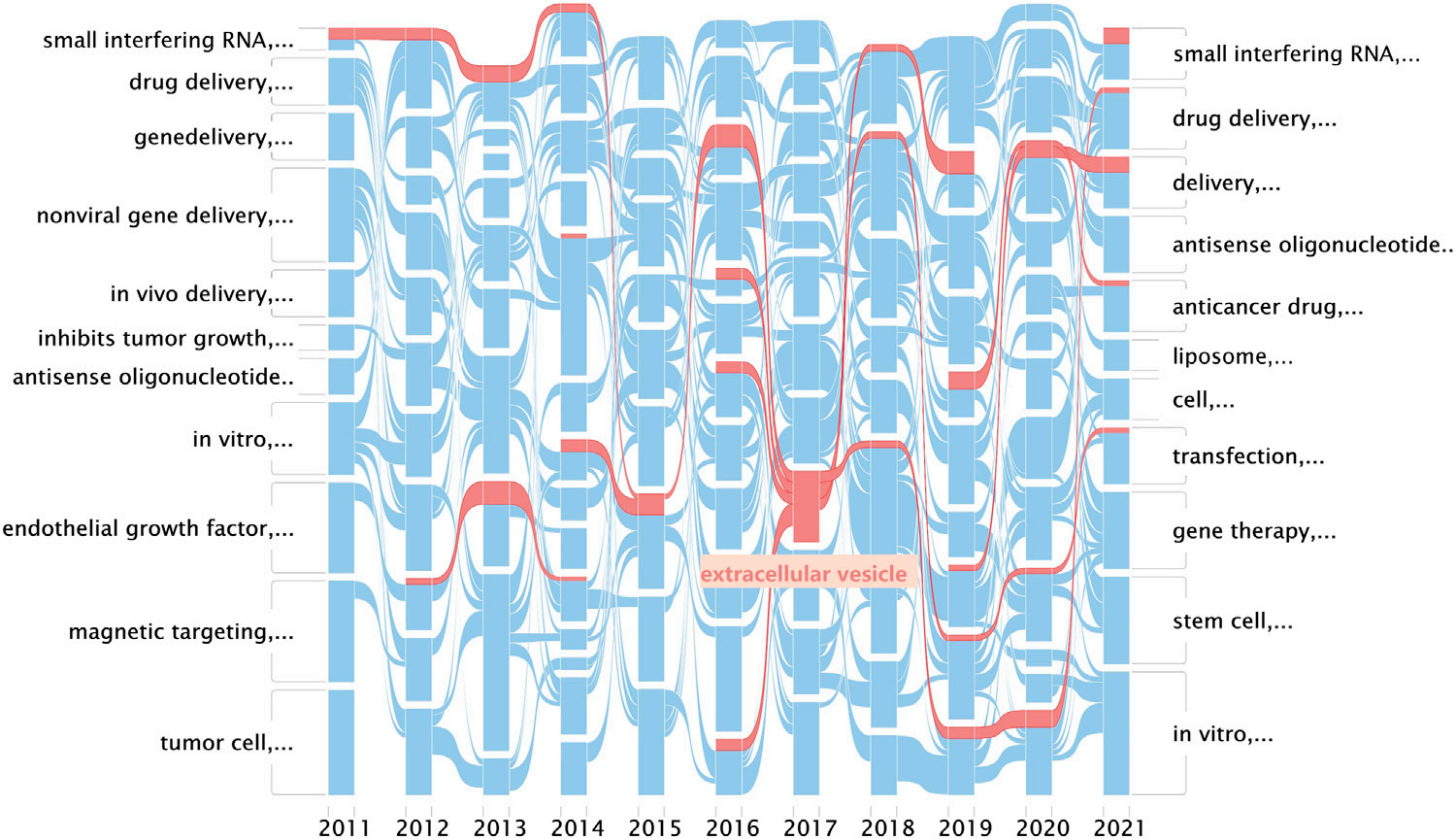
Alluvial flow diagram of nanoparticles in nucleic acid–targeted delivery. Networks are clustered and simplified by year, and streamlines connect the same nodes between years. Every row in this figure represents a keyword co-occurrence network corresponding to the sliced year; blocks refer to clusters of words and are named by the most crucial keyword in each cluster; and the lines connect the same keyword in separated years to observe changes in the concepts over time.

**FIGURE 5:**
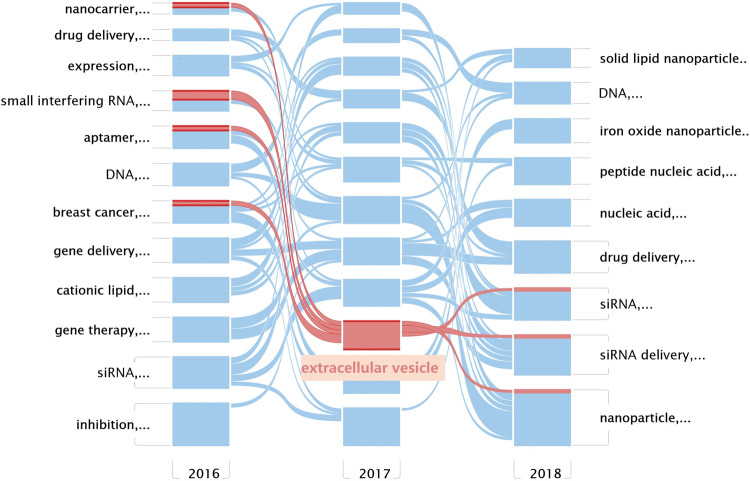
Details of the cluster extracellular vesicle and its connections.

The alluvial generator works as follows: 1) finds clusters, 2) names the clusters by its most important keyword, and 3) displays the clusters by year and connects the same nodes in clusters with streamlines (Bohlin et al., 2014). An alluvial flow diagram reflects the flux of concepts where “blocks” refer to “clusters” of words and are named by the most crucial keyword in each cluster, and the lines connect the same keyword in separated years to observe changes in the concepts over time. As the research field of exosomes is impressive, as shown in Figure 3, the related cluster extracellular vesicle is highlighted in red (Figures 4,5).

## Discussion

A crucial characteristic to discover the landmarks of a network in VOSviewer is high density, which depends on the quantity and quality of the neighboring nodes (van Eck, 2021). This algorithm detects hub nodes, namely, keywords those co-occurred with more different words. As shown in one of the high-density cores in Figure 2, the low transfection efficiency of the nanoparticle-targeted delivery systems has puzzled scientists for a long time (Wilhelm et al., 2016; Chernousova and Epple, 2017). Although no nanoparticles have been presented as a desirable solution for clinical use even to date, several delivery systems are undergoing phase II/III clinical trials, and the SVA of our study has also revealed efforts on the modifications based on polycations, polymers, gold nanoparticles, and lipids regarding the delivery efficiency of siRNA and pDNA (Xue et al., 2013; Ganbold et al., 2017; Herrera et al., 2018; Kwon et al., 2020; Pedziwiatr-Werbicka et al., 2021).

The factors those form an efficient transfection include but are not limited to high stability in circulation, easy escape from endosomes, and rapid release of the cargo (into the nucleus or cytoplasm). The high positive charge density of the polycations promises their affinity to nucleic acids, allows relative success in the endosomal escape likely through the proton sponge effect, but also leads to poor stability for interacting with the serum components and cytotoxicity. Our SVA result highlighted the research in modifying the cationic nano vectors by crosslinking the primary amines of branched polyethylenimine using dimethyl suberimidate. The primary amines were then converted to amidine functions, which were supposed to imitate the arginine-rich motifs of the protein transduction domain to improve internalization efficiency, disperse the charge to reduce toxicity, and expedite intracellular release. The hydrophobicity of the C-8 linker also formed a synergetic membrane penetration effect. All of these possible mechanisms were adequately embodied in the improvement of transfection efficiency both *in vivo* and *in vitro* and the shift of safety in contrast to the native branched polyethylenimine and commercial reagents for transfection (Tripathi et al., 2013).

For the electrically neutral polymers, the clearance effect of the negatively charged glomerular basement membrane and aggregation caused by charged proteins in the blood were no longer the main concerning issues, but slow unpacking of cargo and entrapment in the endosomes made them uncompetitive (Yin et al., 2014). The SVA also detected several remodels in such nanoparticles, typically, the peptide spiders presented with a high MCR (Table 2). The polyethylene glycol (PEG) acted as an octo-valent backbone and was functionalized by the tumor-targeted peptide iRGD and membrane-active peptide transportan in reducible or non-reducible linkages. The hydrophilic shielding effect of PEG maintained the stability of nanocarriers, specifically binding between iRGD and α_v_β_3_ integrins, an upregulated marker on neoplastic tissues, achieved an accurate delivery toward tumor cells and stroma, and transportan, of which the mechanism remained unclear, was speculated to interact with the cell surface by electrostatic/hydrophobic ions or hydrogen bonding upon their structure, hence triggering internalization *via* endocytosis or/and direct translocation (Kwon et al., 2020; Langel, 2021). The microscopic images presented endosomal escape of siRNA in the peptide spiders with reducible linkages intuitively, and the peptide spiders showed an efficiency of ∼50% in silencing the targeted gene, lower than commercial reagents, but successfully maintaining a higher cell viability (>80%) *in vitro* (Kwon et al., 2020). The peptide spiders linked with iRGD and transportan might be a solution to the PEG dilemma, and other researchers have highlighted similar modifications to PEG (Fang et al., 2011).

To further understand the mechanism of the nanoparticles delivery system, special detection methods focusing on different transfection steps are urgently needed, and there have been some exciting attempts recently (Verrneulen et al., 2018; Jiang et al., 2020; Kim et al., 2021).

The keywords tumor cell, nanotechnology, and membrane antigen constitute another core of the density map. 67.4% of the gene therapy clinical trials addresses cancer diseases as their indication^3^. Tumors feature an abnormal growth of cells, and malignant tumors are still a common cause of disease-related deaths. In order to fight cancer, patients receive surgery, radiotherapy, chemotherapy and other treatments, at the cost of aesthetic changes, systemic derangement and functional loss. Comparatively, targeted gene therapy offers a competitive choice to repair mutated genes or eliminate the influence, and the permeability and retention effect makes solid tumors a perfect target in drug delivery systems of nanoparticles (Maeda, 2012). Nanotechnology is widely used to deliver hereditary substances against serum endonucleases, immune detection, and renal clearance, and there were significant achievements in which the aptamers stood out from the others in targeting delivery (Yin et al., 2014; Pereira-Silva et al., 2020). Aptamers are oligonucleotides with specific three-dimensional structures that bind to targeted receptors (Zhou and Rossi, 2017). In the co-citation network, clusters #4 and #10 (aptamer) were close to the #16 RNA-bioconjugates (Figure 3), which indicates that the aptamers have been widely used in coupling with RNA for targeted delivery. SELEX (systematic evolution of ligands by exponential enrichment) is a technology to select high-affinity aptamers *in vitro* (Zhou and Rossi, 2017). Different SELEX approaches have been developed for higher aptamer specificity and a faster selection cycle (Zhang et al., 2019). In the density map, SELEX was in close quarters with the keyword membrane antigen (Figure 2). Cell-specific membrane antigens are ideal binding sites to realize the targeted delivery of the aptamer-conjugated nanoparticles, although adjustments of carriers are required to improve targeting efficiency, loading ability, the half-lifespan, and binding kinetics (Zhou and Rossi, 2011). The introduction of locked nucleic acid enhanced aptamer’s serum stability and this hybrid has been applied in research of delivering therapeutics targeting colon cancer (Roy et al., 2015a; Roy et al., 2015b).

An abnormal citation sometimes announces change in the domain of science, and unusually, it is in cluster #17 (genome editing) for mismatching in early publishing and delayed citing (Table 1). Unlike RNA, the delivery of genome editing therapeutics involves large cargo capacity, off-target toxicity, and immune reaction, and the non-virus vector might be a good answer (van Haasteren et al., 2020). Genome editing operations were greatly simplified by the targeted specificity formed from CRISPR–Cas9 (clustered regularly interspaced short palindromic repeats- CRISPR-associated protein 9) through a very short RNA coding region since its discovery in 1987. CRISPRs are repeated segments of DNA found initially in prokaryotic organisms, and the Cas proteins are endonucleases that use single guide RNA (sgRNA) to form a complementary base pair with the target DNA and then cut the DNA at a specific site (Ishino et al., 1987; Liu et al., 2017). Among them, Cas9 has been widely used because of its efficiency and simplicity (Liu et al., 2017). In recent years, the safety and efficiency shown in clinical trials reduced the concerns about gene therapy, which was sufficiently certified by citing the events of cluster #17 (Naldini, 2015). Therefore, CRISPR–Cas9 may open new avenues for cancer research and treatment, and further exploration of the nanocarriers to introduce CRISPR–Cas9 for both *in vivo* and *in vitro* gene therapy is needed.

Depending on the purpose of gene editing, CRISPR-Cas9 is used to mediate gene knockout *via* nonhomologous end joining, or precisely editing genes by homology-directed repair, and the requisite elements in the former situation are Cas9 protein and sgRNA, while for precise editing, a supernumerary donor template is also required (Cong et al., 2013). The cargo of this system can be: 1) pDNA that expresses both sgRNA and Cas9 protein; 2) sgRNA and mRNA of Cas9 protein; 3) sgRNA and Cas9 protein; or 4) donor DNA when necessary. The deliberate design of the nanocarriers for different loadings is critical to effective delivery and appropriate unpacking in certain subcellular localization (Liu et al., 2017).

Remarkably, in both the mean publication year (2015) and citing year (2020), our research suggested that the exosomes were likely another hotspot of nanomedicine in the targeted delivery of nucleic acids, which made cluster #1 exosomes look “young” as shown in Figure 3 (Di et al., 2018). Exosomes, a family of extracellular vesicles, with diameters ranging from 40 to 130 nm, are communicators of intercellular information. As shown in Figures 4, 5, studies related to the extracellular vesicles can be traced back to 2011 in the co-occurrence network but did not form a cluster until 2017 (Alvarez-Erviti et al., 2011). Using the exosomes to deliver siRNA has been an attractive choice for their natural ability to load, sort, and package biomacromolecules, cross physical barriers (especially the blood–brain barrier), and safety (Elsharkasy et al., 2020). Several ways to functionalize the exosomes with aptamers have been achieved to inhibit the growth of cancer cells; in addition, aptamers can also mediate selective DNA assembly on the membrane surface of the exosomes (Wan et al., 2017; Pi et al., 2018; Tran et al., 2020). As the exosomes derived from autologous breast cancer cells had a high affinity to the lungs, the exosomes’ membrane helped nanoparticles load therapeutic siRNA targeted to the pre-metastatic niche of the lungs, thus restraining the metastasis of breast cancer (Zhao et al., 2020). Despite low yield, instability, and unclear mechanism cramping the progress of translation, a series of proposals on standardized manufacture make the exosomes viable nanoparticles in nucleic acid delivery (Buschmann et al., 2021; Butreddy et al., 2021).

There are also limitations in our study. First, on account of the best performance CiteSpace, choosing the Web of Science as the only source of our research implied possible bias in the absence of literature that were only included by other databases. Besides, applying different computation models in bibliometrics may slightly affect the outcomes, although we have made our best effort to optimize software selection and interpret the results. In addition, our purpose was to detect deeply into the science domain of nanomedicine on the targeted delivering of nucleic acids. Thus, the collaboration networks were not presented in this study. In fact, defining impactive authors, institutions, and countries in this field matters a lot in allocating research finance. Furthermore, since its significant advantage in contrast to viral vector is safety, the efficiency of the nanocarrier is emphasized more in this bibliometric study based on existing materials, although effectiveness and safety share equivalent significance virtually. Last but not least, the limitation of our topic resulted in the absence of presenting research advances on the viral delivery platform in our study; in fact, experiences in translating viral vectors to the clinic in gene therapy are worth studying.

In summary, our study depicted the landscape in the scientific domain of nanoparticles targeted delivering of nucleic acids by co-occurrence and co-citation networks, where the low transfection efficiency was a major task to overcome. The SVA revealed representative modifications on polycations, polymers, gold nanoparticles, and lipids, but special detection methods for transfection are urgently needed. SELEX-enriched aptamers play an essential role in RNA targeted delivery. Specially, the co-citation analysis highlighted the exosome as a promising vector. Besides, integrating nano vectors with gene editing platforms has become an exciting research tendency. Through the above analysis, we aimed to establish a general knowledge system for biologists, chemists, and pharmacists interested in this field and offer visualized and valuable information for policy decisions to promote translations.

## Retrieval Strategy 1

TS = (“Nanoparticles” OR “Nanoparticle” OR “Nanocrystalline” OR “Nanocrystalline Material” OR “Nanocrystal” OR “nanoparticles” OR “nano particle” OR “nano particles”) AND TS = (“Nucleic Acids” OR “Nucleic Acid” OR “Nucleotide” OR “Nucleotides”) AND TS = (“Targeted” OR “Targeting” OR “target”) AND TS = (“Therapeutic” OR “Therapy” OR “Therapies”).

## Data Availability

The original contributions presented in the study are included in the article/Supplementary Material, further inquiries can be directed to the corresponding author.

## Footnotes

1
https://a873679.fmphost.com/fmi/webd/GTCT, accessed 27 March 2022.

2
https://www.mapequation.org/apps/MapGenerator.html, accessed 27 March 2022.

3
https://a873679.fmphost.com/fmi/webd/GTCT, accessed 27 March 2022.

## Abbreviations

A&HCI, arts & humanities citation index; CCR-EXPANDED, current chemical reactions; CPCI-S, conference proceedings citation index—science; CPCI-SSH, conference proceedings citation index—social sciences & humanities; CRISPR–Cas9, clustered regularly interspaced short palindromic repeats–CRISPR-associated protein 9; ESCI, emerging sources citation index; IC, index chemicus; MCR, modularity change rate; PEG, polyethylene glycol; Q, modularity; SCI-EXPANDED, science citation index expanded; SELEX, systematic evolution of ligands by exponential enrichment; sgRNA, single guide RNA; SSCI, social sciences citation index; SVA, structural variation analysis.
