# Supplementary material for: Trends and Hotspots in Nanoparticles for the Targeted Delivery of Nucleic Acids: A Ten-Year Bibliometric Study
Source: Front Pharmacol. 2022 May 4;13:868398. doi: 10.3389/fphar.2022.868398 (PMC9114467; doi:10.3389/fphar.2022.868398)
Supplement: Supplementary file 1 [file Table1.docx]

Supplementary Material

# Supplementary Table

**Table S1.** Cited references and citing articles of Cluster #17 genome editing.

| **Cited references** | | **Citing articles** | |
| --- | --- | --- | --- |
| **Year** | **Title** | **Year** | **Title** |
| 2017 | Delivery technologies for genome editing | 2021 | Rational designs of in vivo CRISPR-Cas delivery systems |
| 2017 | CRISPR/Cas9-Based Genome Editing for Disease Modeling and Therapy: Challenges and Opportunities for Nonviral Delivery | 2021 | Strategies in the delivery of Cas9 ribonucleoprotein for CRISPR/Cas9 genome editing |
| 2017 | Nanoparticle delivery of Cas9 ribonucleoprotein and donor DNA in vivo induces homology-directed DNA repair | 2021 | Recent advances in chemical modifications of guide RNA, mRNA and donor template for CRISPR-mediated genome editing |
| 2015 | Cationic lipid-mediated delivery of proteins enables efficient protein-based genome editing in vitro and in vivo | 2021 | In vivo targeted delivery of nucleic acids and CRISPR genome editors enabled by GSH-responsive silica nanoparticles |
| 2015 | Self-Assembled DNA Nanoclews for the Efficient Delivery of CRISPR–Cas9 for Genome Editing | 2021 | Evolutionary timeline of genetic delivery and gene therapy |
| 2014 | The new frontier of genome engineering with CRISPR-Cas9 | 2021 | A nucleic acid/gold nanorod-based nanoplatform for targeted gene editing and combined tumor therapy |
| 2013 | Multiplex Genome Engineering Using CRISPR/Cas Systems | 2021 | Membrane-destabilizing ionizable phospholipids for organ-selective mRNA delivery and CRISPR-Cas gene editing |
| 2012 | A Programmable Dual-RNA–Guided DNA Endonuclease in Adaptive Bacterial Immunity | 2021 | Recent development of gene therapy for pancreatic cancer using non-viral nanovectors |
| 2002 | Identification of genes that are associated with DNA repeats in prokaryotes | 2020 | The delivery challenge: fulfilling the promise of therapeutic genome editing |
